# Monitoring Xenon Capture in a Metal Organic Framework Using Laser-Induced Breakdown Spectroscopy

**DOI:** 10.3390/mi14010082

**Published:** 2022-12-29

**Authors:** Hunter B. Andrews, Praveen K. Thallapally, Alexander J. Robinson

**Affiliations:** 1 Oak Ridge National Laboratory, Oak Ridge, TN 37830, USA; 2 Pacific Northwest National Laboratory, Richland, WA 99352, USA

**Keywords:** laser-induced breakdown spectroscopy (LIBS), metal organic framework (MOF), noble gases, radionuclides, fission gases, selectivity, breakthrough tests, off-gas sensor

## Abstract

Molten salt reactor operation will necessitate circulation of a cover gas to remove certain evolved fission products and maintain an inert atmosphere. The cover gas leaving the reactor core is expected to contain both noble and non-noble gases, aerosols, volatile species, tritium, and radionuclides and their daughters. To remove these radioactive gases, it is necessary to develop a robust off-gas system, along with novel sensors to monitor the gas stream and the treatment system performance. In this study, a metal organic framework (MOF) was engineered for the capture of Xe, a major contributor to the off-gas source term. The engineered MOF column was tested with a laser-induced breakdown spectroscopy (LIBS) sensor for noble gas monitoring. The LIBS sensor was used to monitor breakthrough tests with various Xe, Kr, and Ar mixtures to determine the Xe selectivity of the MOF column. This study offers an initial demonstration of the feasibility of monitoring off-gas treatment systems using a LIBS sensor to aid in the development of new capture systems for molten salt reactors.

## 1. Introduction

Molten salt reactors (MSRs) are an advanced nuclear reactor design in which the working fluid is a liquid salt, and the fuel is either solid or dissolved into the liquid salt. Typically, these salts are either fluorides or chlorides depending on whether the reactor design is a thermal or fast spectrum reactor. MSRs are being pursued because of several inherent advantages over traditional light water reactors, including passive safety features, high exergy, high thermodynamic efficiency, and the potential for greater fissile material utilization [1,2]. This later benefit comes from the capability to perform real-time fission product removal or online reprocessing. Some level of this removal occurs passively as gaseous and volatile fission products leave the salt. 

Although this removal is beneficial for reactor neutronics because fission products with large neutron absorption cross sections leave the salt, it generates another challenge in that these fission gases need to be properly captured in an off-gas system that acts as a radionuclide boundary and release prevention system. An off-gas system will contain multiple components to treat different aspects of the cover gas coming from an MSR core. These components can include a decay tank for short-lived radioisotopes to decay; a molten hydroxide scrubber for particulate and acidic gas removal; halide, H_2_O and O_2_ traps; and activated charcoal for noble gas delay [1,2]. There is also the potential for noble gas capture with methods such as cryogenic distillation, which would have a potential commercial value [1,2]. The treated gas stream would then be resupplied to the core as the regenerated cover gas. 

The noble gases produced through fission or subsequent decay make up a large contribution of the source term anticipated in the off-gas stream. During the Molten Salt Reactor Experiment, activated charcoal was used to hold up these gases long enough for the isotopes to decay. The isotopes of Xe and Kr have a large range of half-lives varying from 39.5 s to 5.25 d and from 32.3 s to 10.76 y for Xe and Kr, respectively. The isotopes with half-lives on the scale of hours and days cause the most trouble in terms of treatment and require a significant residence time in the charcoal beds (e.g., 90 d). This requires a large footprint with four to five charcoal beds 6–9 ft in diameter and 50 ft in length [2]. 

An alternative technology to charcoal delay beds is metal organic frameworks (MOFs). MOFs are highly porous crystalline materials composed of metal ions linked by organic molecules. The combination of metal ions and organic linker molecules allows these materials to be engineered for specific gas selectivity. These porous structures have several advantages compared with charcoal delay beds, including a significant cost reduction from a higher selectivity and capacity, smaller size, and posing no fire hazard. Additionally, Xe and Kr can be captured in separate steps, which might permit the gases to be sold for their commercial value. 

In addition to the need to develop more efficient off-gas treatment components, there is a need to develop sensors to be used in-line with these components that can monitor species’ concentrations. The mixed gas and particulate stream, anticipated radiation field, and breadth of analytes makes monitoring an MSR off-gas system a challenge for traditional approaches. Optical spectroscopy is ideal for online monitoring because it is sensitive to a large array of analytes, can be deployed remotely using optical fibers, and can monitor both stable and radioactive species. Laser-induced breakdown spectroscopy (LIBS) has recently been demonstrated to be capable of monitoring both aerosolized species and noble gases in real time [3,4]. A sensor such as this would be a valuable tool to be coupled with noble gas treatment components being evaluated on an MSR off-gas testbed. With continued improvement in pulsed laser systems, LIBS sensors can be reduced in size and more readily deployed in the field.

The goal of this study was twofold: (1) develop sorbents and fabricate them into an engineered form to capture noble gases at or near room temperature and (2) develop and test an optical spectroscopy sensor to monitor noble gas capture. These activities demonstrated the use of optical spectroscopy coupled with sorbent materials to capture and monitor the noble gases in real time and resulted in an engineered gas treatment testbed for future component development and evaluation.

## 2. Experimental

### 2.1. MOF Synthesis and Packing

Among all the MOFs tested in a recent study, the calcium-based MOF, CaSDB (SDB = 4,4′–sulfonyldibenzoate), with a pore diameter of 4.5 Å was shown to outperform all the materials tested at room temperature [5,6,7,8,9]. Based on this, a CaSDB MOF was prepared for this study. The CaSDB was synthesized under solvothermal conditions from a stoichiometric solution of SDB, and CaCl_2_•2H_2_O. The synthesis procedure was slightly modified from published procedures, where equimolar reagents were used, in an attempt to produce more CaSDB [10]. In this study, a 2 L glass liner was charged with 1.0 L of EtOH, 16.45 g of SDB (53.7 mmol), and 8.48 g of CaCl_2_•2H_2_O, (57.7 mmol) then sealed in a 2 L stainless steel Parr reactor. The vessel was heated to 180 °C for 48 h to yield an off-white crystalline powder. The powder was washed with acetone (3 × 35 mL) and collected by filtration. To prepare engineered particles of CaSDB, powdered CaSDB was packed into a silicon tube and pressed at 2000 psi (13.8 MPa) for 3 min using an isostatic press to obtain CaSDB pellets. The pellets were carefully broken up, and the fragments were sieved for 600–850 μm sized particles. The purity of the produced MOF was evaluated using X-ray diffraction (XRD) and single component gas adsorption tests, and then 1 g of engineered particles were packed in a stainless-steel tube and sent for LIBS testing.

### 2.2. LIBS and MOF Breakthrough Testing

**Figure 1:**
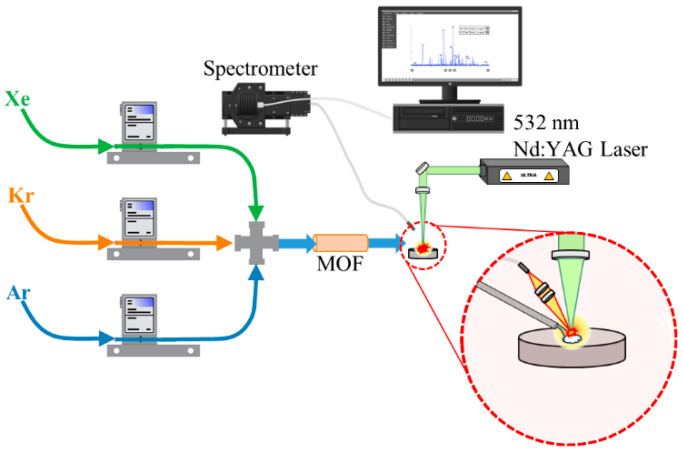
Experimental schematic for LIBS gas analysis with MOF filter.

A custom gas testing system was built to use LIBS to monitor noble gas transfer through various filter materials with different gas mixtures. A three-way gas manifold system was configured with mass flow controllers (Sierra Instruments, Monterey, CA, USA, SmartTrak100) calibrated for Ar, Kr, and Xe gas flows up to 3 mL min^−1^. Each line was attached to compressed cylinders with pure source gasses (AirGas, Oak Ridge, TN, USA, 99.99%). Following the junction after the mass flow meters, the gas line was plumbed into a laser enclosure for LIBS measurements. The gas line was delivered to the sampling point using a pipette tip as a nozzle aimed toward the surface of an aluminum substrate. A 532 nm Nd:YAG laser (Lumibird, Bozeman, MT, USA, Ultra) was fired and focused onto the aluminum substrate. The substrate was used to ensure breakdown occurred at a repeatable position as air breakdown did not occur with the 532 nm laser and energy ranges used. The height of the aluminum substrate was aligned with the laser focal point using a Keyence laser to measure distance. The plasma light was collected at ~45° from the laser pulse and was routed to the spectrometer using a 2 m fiber-optic cable (Avantes, Lafayette, CO, USA, FC-UV200). An echelle-type spectrometer (Catalina Scientific, Tucson, AZ, USA, EMU-120/65) was used along with an electron multiplying charged-coupled device (Raptor Photonics, Falcon Blue) and a pulse generator for external triggering (Quantum Composers, Model 9214). The spectrometer was wavelength calibrated using a Hg:Ar lamp (StellarNet Inc., Tampa, FL, USA, SL2). A diagram of the planned experimental setup is shown in Figure 1. 

Three breakthrough tests were completed to investigate Xe capture by the engineered MOF column. Before the breakthrough tests, the MOF column was activated in a vacuum oven at 100 °C for 12 h to remove any absorbed gases. The MOF was then connected to the test system. The mass flow controllers were used to adjust the gas stream composition to the desired Xe and Kr ratios (the balance was Ar) at a flow rate ranging from 23–28 mL min^−1^. While bypassing the MOF, this gas stream ran for 10 min to establish an equilibrium. Then, the LIBS instrument was initiated and run for 2 min before the bypass was switched to direct gas flow through the MOF column. This time was recorded and used as the time zero for determining breakthrough times. These tests were run for a minimum of 30 min, and the laser was operated at 2 Hz.

### 2.3. Optimization of LIBS Acquisition Settings

LIBS measurements are subject to the acquisition settings used when forming the plasma and observing it. These settings include spectrometer delay and exposure time, laser energy, and the number of shots used. These parameters were optimized by investigating the signal response to changes through tracking the signal-to-background ratio (SBR) [11]. The SBR is defined in Equation (1). The system settings were optimized using the pure Ar gas and the 763.5 nm Ar I peak to conserve the noble gas stocks.

(1) $$\mathrm{SBR}=\frac{\mathrm{Emission}\;\mathrm{peak}\;\mathrm{intensity}}{\mathrm{Nearby}\;\mathrm{baseline}\;\mathrm{intensity}}$$

**Figure 2:**
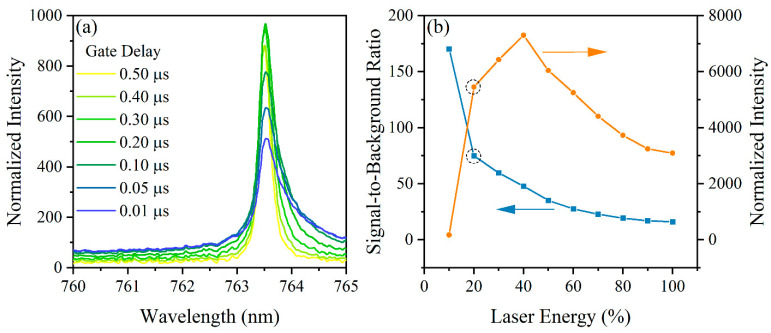
Spectral response of the 763.5 nm Ar I peak versus (**a**) gate delay and (**b**) laser energy. The signal-to-background ratio and normalized peak intensity shown in (**b**) were both considered when selecting the optimal collection settings to be 0.5 µs delay and 20% laser energy.

First, the spectrometer gate delay was optimized; the delay is the time between firing the laser pulse and the spectrometer initiating light collection. The impact of the delay time on the peak emission is shown in Figure 2a. As the delay time is increased, the peak grows in intensity and the background levels fall. The background levels fall as the result of having less white light from the plasma continuum emissions when observing later in the plasma lifetime. The optimal gate delay was determined to be 0.5 µs. The spectrometer used in this study did not have an internal shutter, meaning there was an effective minimal exposure time. The exposure time was set to 200 µs to collect data as fast as possible with the available spectrometer.

The laser pulse energy and signal intensity are directly linked through the ablation process. The laser power density impacts the peak plasma temperature and electron density. Plasma temperature and electron density affect the relative ratios of the species’ ionization levels in the plasma, as well as the transition rates (i.e., intensities) of species. The laser used in this study had an upper energy limit of 50 mJ pulse^−1^. The response of the argon peak intensity to changes in the laser energy are shown in Figure 2b. The laser energy was varied from 10% to 100% by adjusting the internal Q-switch delay. An adequate plasma was always formed at laser energies 20% and greater, but the plasma formation at 10% was inconsistent. To optimize the energy setting, not only was the SBR taken into consideration but also the signal intensity itself was given weight. If only the SBR is considered, then 10% energy would be the optimal value; however, as mentioned previously, plasma formation was a challenge at this level. The SBR at 10% energy is so large primarily because the background intensity levels are almost nonexistent. To determine the optimal energy, each setting was given percentile ranks for their SBR and intensity values. These ranks were then factored together to provide an overall score, which indicated 20% energy as the optimal balance of high SBR and large signal intensity. This corresponds to a pulse energy of approximately 10 mJ.

## 3. Results and Discussion

### 3.1. Preliminary MOF Analysis

**Figure 3:**
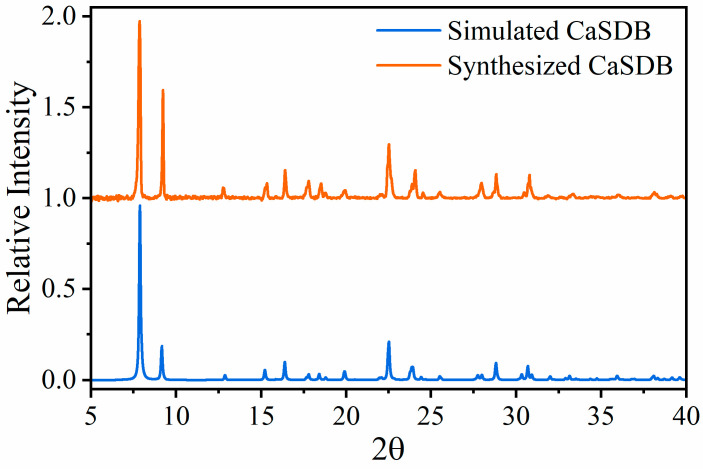
Simulated and experimental XRD measurements of synthesized CaSDB powder.

XRD measurements were used to analyze the phase composition and structure of CaSDB powder and the CaSDB engineered particles. The sample was placed in a powder sample holder under ambient conditions, and an XRD pattern was collected continuously using CuKα1 radiation (λ = 1.54056, 2 θ range from 5° to 40°). The bulk sample XRD signal was found to be identical to the simulated XRD signal, suggesting the phase purity of the CaSDB. The XRD measurement of the synthesized CaSDB compared to the simulated pattern is shown in Figure 3. 

**Figure 4:**
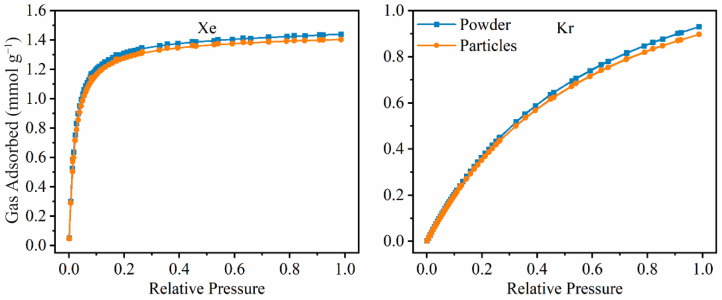
Noble gas adsorption and desorption at room temperature using CaSDB MOF powder and engineered particles.

To further evaluate the CaSDB purity and structural integrity, single component gas adsorption analysis was performed using an in-house gas adsorption analyzer. The sample was activated at 100 °C under vacuum at a rate of 5 °C min^−1^ on the outgassing side of the instrument. The sample was then cooled to room temperature, and the dry mass was measured. The experimental temperature of 25 °C was maintained by a water bath. The pressure points were set beforehand using the software. Volumetric changes, resulting from adsorption at each pressure step, were plotted against the pressure. The pure-component xenon adsorption isotherms at room temperature were performed to demonstrate the purity of the MOF powder and engineered particles. Both forms of MOF samples saturate quickly (at 0.2 bar), which is indicative of a strong framework-Xe interaction. At room temperature and 1 bar, the MOF adsorbs 1.3 mmol g^−1^ of Xe, whereas under the same conditions, Kr adsorption is 0.8 mmol g^−1^. However, the engineered particles have a slightly lower Xe and Kr adsorption capacity at all the pressure points (Figure 4). Similar adsorption isotherms for other gases including Ar, N_2_, and O_2_ have been conducted on CaSDB in previous studies [10]. These gasses all show delayed saturation indicating the impacts of Ar being used as the matrix gas in the breakthrough tests was negligible. The XRD coupled with pure component gas adsorption data on the CaSDB MOF suggest the phase purity of the synthesized material was adequate.

### 3.2. LIBS System Testing

**Figure 5:**
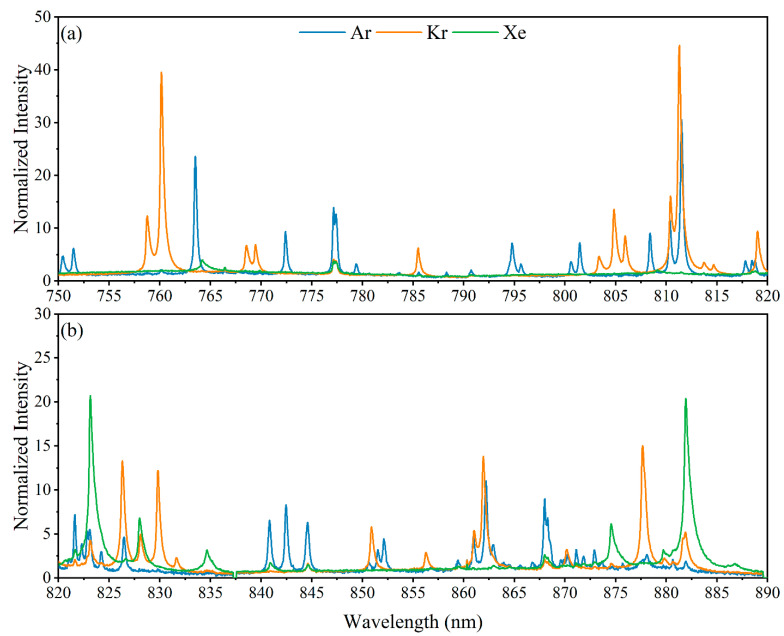
LIBS emissions of Ar, Kr, and Xe gasses overlaid to illustrate peak interferences in relevant wavelength ranges (**a**) 750–820 and (**b**) 820–890.

Before performing any breakthrough tests on the MOF column, the three stock noble gases were measured with LIBS to benchmark their associated elemental fingerprints. The overlaid spectra of the three noble gases are shown in Figure 5 in the near infrared range from 750 to 890 nm, where the gas emissions dominate the spectra. Over the 140 nm wavelength range shown are several unique peaks for Ar and Kr. Xenon, however, has far fewer strong emissions, and several of them are convoluted with other species’ emissions. Fortunately, the four strongest Xe emission peaks at 823.15, 834.67, 875.56, and 881.95 nm have little interference or have a far larger relative intensity than their interferent peaks. A handful of other emission peaks of relevant species are seen outside the spectral range plotted in Figure 5.

The spectra produced using LIBS can vary due to shot-to-shot fluctuations in laser energy, variations in the plasma formation, or slight changes in the substrate composition. To help overcome these effects, multivariate models are typically constructed using partial least squares regression (PLSR). This technique iteratively identifies variables in a latent space that explain the most variance in the Y matrix (concentration). These latent variables are then used to regress the raw signals into a concentration prediction. PLSR has been used frequently for quantifying optical spectroscopy signals and the mathematics of this approach are discussed elsewhere [12,13,14,15].

**Figure 6:**
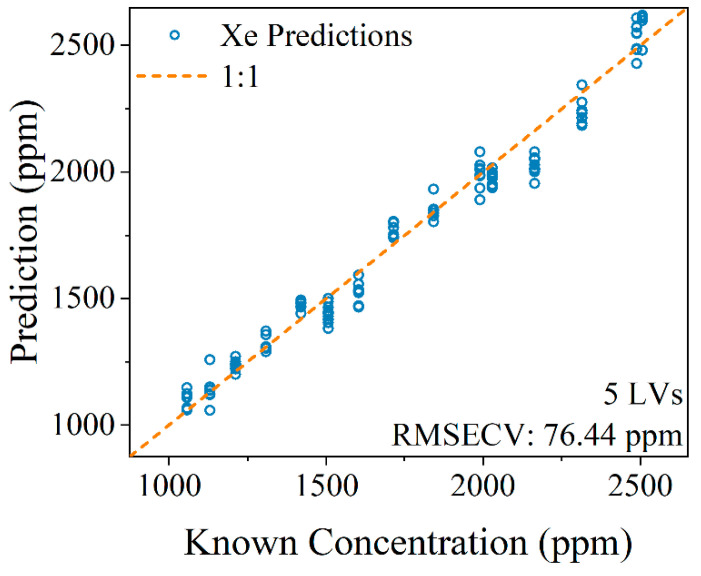
Parity plot comparing known Xe concentrations to those predicted by a multivariate partial least squares model. If a point falls on the dashed 1:1 line it represents a perfect prediction.

Here, a PLSR model was built on the spectral region shown in Figure 5 (750–890 nm) using 4500 spectra of gas mixtures ranging from 1000 to 2500 ppm Xe. The spectra were normalized to the maximum intensity of each spectrum (Ar I 763.5 nm), and then every 20 spectra were averaged. The dataset was split 50:50 into training and test datasets. The PLSR model showed strong prediction performance on the test set with a root mean squared error of cross validation of 76.44 ppm. These predictions are illustrated in the parity plot shown in Figure 6. For the subsequent breakthrough tests only qualitative measurements were needed, but this multivariate model was built to evaluate the limits of detection of the constructed system. A pseudounivariate approach was used to calculate the limits of detection based on how well the model predicts the samples used to construct it [16]. The pseudounivariate limit of detection for Xe was 167 ppm. This value serves as a conservative estimation of the system’s sensitivity. This value confirmed that the LIBS system would be adequate for sensing Xe passing through the MOF. 

### 3.3. Breakthrough Analysis via LIBS

**Figure 7:**
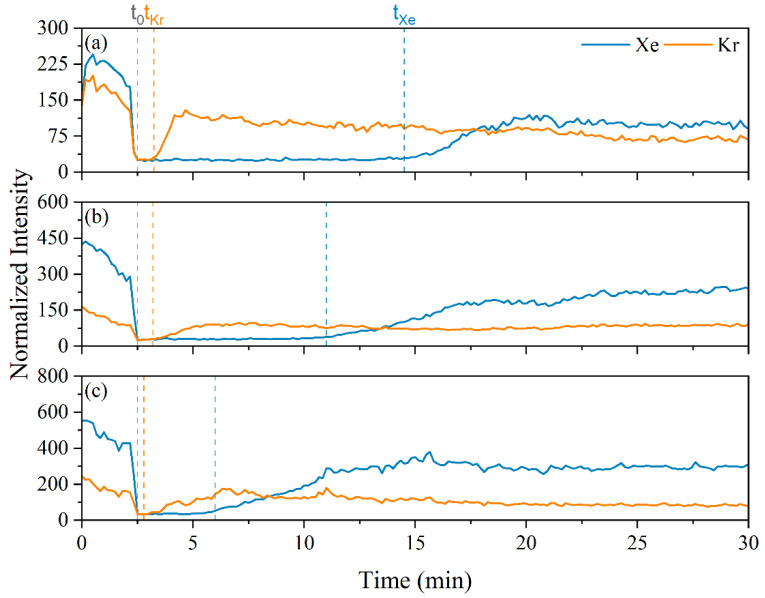
Xe and Kr breakthrough curves for various mixture ratios: (**a**) 1000 ppm Xe, 1000 ppm Kr, (**b**) 2000 ppm Xe, 1000 ppm Kr, and (**c**) 3000 ppm Xe, 1000 ppm Kr.

Following the preliminary MOF characterization and LIBS system testing, the MOF column was placed in line with the mass flow controllers and the LIBS system for breakthrough tests. Three breakthrough tests were performed with following gas ratios: 1000 and 1000 ppm, 2000 and 1000 ppm, and 3000 and 1000 ppm Xe and Kr, for tests 1–3, respectively. The breakthrough curves for Xe and Kr in the three tests are shown in Figure 7.

The breakthrough times correspond to the time between the gas concentrations going to zero and when the gas concentrations begin to increase, indicating the species passing through the column to the measurement point. The From the breakthrough tests shown in Figure 7, the Xe breakthrough time was reduced as the Xe concentration in the gas stream increased. Intuitively, the Xe peak intensities increased from test to test as the Xe gas concentration was increased. Similarly, the Kr breakthrough times, and peak intensities were consistent across the tests as Kr concentration was held constant. The Xe breakthrough times were 12, 8.5, and 3.5 min. The Kr breakthrough times were 0.75, 0.70, and 0.30 min. These breakthrough times can be used with the gas compositions to determine the Xe and Kr loading on the MOF and then the MOF selectivity can be calculated using Equation (2):

(2) SXe/Kr=xXeyXexKryKr

where *x* corresponds to the mole fraction absorbed by the MOF and *y* corresponds to the mole fraction in the bulk gas [17].

The selectivity of the MOF was calculated to be 16, 12, and 12 for tests 1, 2, and 3, respectively. These values are on par with those reported previously for other Xe selective MOFs [10,18]. The decrease in selectivity from test 1 to the subsequent tests may be due to effects from multiple use and regeneration, slight changes in overall flow rates, or the release of adsorbed gases during the activation process not being entirely complete. Regardless, the measurement capabilities have been demonstrated.

## 4. Conclusions

Advanced reactors offer many benefits and simultaneously pose many challenges. The proper treatment of the produced radiological off-gas stream is essential for the continued development of MSRs. More modern approaches to gas capture, such as MOFs, provide new flexibility to selectively remove gases, but these methods will need to be proven at an engineering scale. To do this, novel monitoring methods that can cope with radioactive and stable species, multiple sample forms (i.e., gases and aerosols), and provide real-time quantification are needed. In this study, we have demonstrated the ability to use LIBS as a sensor to monitor the capture of gases in near-real time. The sensitivity and the time resolution of this LIBS sensor can be enhanced by using a higher frequency laser. Future work will also include the removal of a sample substrate to shoot the gas stream directly. This will enable the testing of treatment components on engineered test systems such as molten salt loops, which will better reflect the conditions of an operating MSR.

## Data Availability

Data is available upon reasonable request to the corresponding author.

## References

[1] Riley B.J., McFarlane J., DelCul G.D., Vienna J.D., Contescu C.I., Forsberg C.W. (2019). Molten Salt Reactor Waste and Effluent Management Strategies: A Review. Nucl. Eng. Des..

[2] Andrews H.B., McFarlane J., Chapel A.S., Ezell N.D.B., Holcomb D.E., de Wet D., Greenwood M.S., Myhre K.G., Bryan S.A., Lines A. (2021). Review of Molten Salt Reactor Off-Gas Management Considerations. Nucl. Eng. Des..

[3] Andrews H.B., Myhre K.G. (2022). Quantification of Lanthanides in a Molten Salt Reactor Surrogate Off-Gas Stream Using Laser-Induced Breakdown Spectroscopy. Appl. Spectrosc..

[4] Andrews H.B., McFarlane J., Myhre K.G. (2022). Monitoring Noble Gases (Xe and Kr) and Aerosols (Cs and Rb) in a Molten Salt Reactor Surrogate Off-Gas Stream Using Laser-Induced Breakdown Spectroscopy (LIBS). Appl. Spectrosc..

[5] Feng X.H., Zong Z.W., Elsaidi S.K., Jasinski J.B., Krishna R., Thallapally P.K., Carreon M.A. (2016). Kr/Xe Separation over a Chabazite Zeolite Membrane. J. Am. Chem. Soc..

[6] Chen L., Reiss P.S., Chong S.Y., Holden D., Jelfs K.E., Hasell T., Little M.A., Kewley A., Briggs M.E., Stephenson A. (2014). Separation of Rare Gases and chiral Molecules by Selective Binding in Porous Organic Cages. Nat. Mater..

[7] Banerjee D., Simon C.M., Elsaidi S.K., Haranczyk M., Thallapally P.K. (2018). Xenon Gas Separation and Storage Using Metal-Organic Frameworks. Chem-Us.

[8] Chakraborty D., Nandi S., Sinnwell M.A., Liu J., Kushwaha R., Thallapally P.K., Vaidhyanathan R. (2019). Hyper-Cross-Linked Porous Organic Frameworks with Ultramicropores for Selective Xenon Capture. ACS Appl. Mater. Interfaces.

[9] Sun Q., Zhu L., Aguila B., Thallapally P.K., Xu C., Chen J., Wang S., Rogers D., Ma S.Q. (2019). Optimizing Radionuclide Sequestration in Anion Nanotraps with Record Pertechnetate Sorption. Nat. Commun..

[10] Banerjee D., Simon C.M., Plonka A.M., Motkuri R.K., Liu J., Chen X.Y., Smit B., Parise J.B., Haranczyk M., Thallapally P.K. (2016). Metal-Organic Framework with Optimally Selective Xenon Adsorption and Separation. Nat. Commun..

[11] Andrews H., Phongikaroon S. (2019). Development of an Experimental Routine for Electrochemical and Laser-Induced Breakdown Spectroscopy Composition Measurements of SmCl_3_ in LiCl-KCl Eutectic Salt Systems. Nucl. Techy..

[12] Beebe K.R., Pell R.J., Seasholtz M.B. (1998). Chemometrics: A Practical Guide.

[13] Bro R. (1996). Multiway Calibration. Multilinear PLS. J. Chemom..

[14] de Jong S., Wise B.M., Ricker N.L. (2001). Canonical Partial Least Squares and Continuum Power Regression. J. Chemom..

[15] Wold S., Hoy M., Martens H., Trygg J., Westad F., MacGregor J., Wise B.M. (2009). The PLS Model Space Revisited. J. Chemom..

[16] Ortiz M.C., Sarabia L.A., Herrero A., Sánchez M.S., Sanz M.B., Giménez D., Meléndez M.E. (2003). Capability of Detection of an Analytical Method Evaluating False Positive and False Negative (ISO 11843) with Partial Least Squares. Chemom. Intell. Lab. Syst..

[17] Liu J., Thallapally P.K., Strachan D. (2012). Metal−Organic Frameworks for Removal of Xe and Kr from Nuclear Fuel Reprocessing Plants. Langmuir.

[18] Fernandez C.A., Liu J., Thallapally P.K., Strachan D.M. (2012). Switching Kr/Xe Selectivity with Temperature in a Metal–Organic Framework. J. Am. Chem. Soc..

